# Benign multicystic peritoneal mesothelioma in a postmenopausal woman complicated with an ovarian cyst: a case report

**DOI:** 10.11604/pamj.2021.40.171.30401

**Published:** 2021-11-19

**Authors:** Eirini-Aikaterini Evangelopoulou, Konstantinos Zacharis, Georgia Skoufi, Nikolaos Vlassis, Papamichali Roidoula, Georgios Lialios

**Affiliations:** 1 Department of Obstetrics and Gynecology, University Hospital of Larissa, Larissa, Thessaly, Greece,; 2 Occupational Health, Private Practice, Larissa, Greece,; 3 Obstetrics and Gynecology, Private Practice, Lamia, Greece,; 4 Department of Pathological Anatomy, University Hospital of Larissa, Thessaly, Greece

**Keywords:** Ovarian cyst, benign, multicystic peritoneal mesothelioma, diagnosis, case report

## Abstract

Benign multicystic peritoneal mesothelioma is a rare cystic neoplasm, characterized by subtle symptoms, that occurs predominantly in reproductive-aged women. The pathogenesis and etiology of the disease are yet to be determined. We herein present a 71-year-old woman presented to our clinic with persistent low back pain. The clinical examination showed a palpable mass in the abdominal area. The magnetic resonance imaging revealed multiple cystic lesions that occupy the largest part of the pelvis, posterior to the uterus. The patient underwent cyst excision, total hysterectomy with bilateral salpingo-oophorectomy, omentectomy and lymph node dissection. Postoperative course was uneventful and histopathology of the specimen revealed a benign multicystic peritoneal mesothelioma. Complete tumor resection is considered the optimal therapeutic approach of peritoneal mesothelioma. Histopathological analysis is required to confirm the diagnosis of multicystic peritoneal mesothelioma.

## Introduction

Benign Multicystic Peritoneal Mesothelioma (BMPM) is an uncommon benign tumor that occurs predominantly in women of reproductive age [1]. The reported prevalence is primarily based on sporadic case reports (fewer than 200 cases) [2]. Peritoneal mesothelioma presents with recurrent peritoneal mesothelial cysts that arise from the epithelial and mesenchymal elements of mesothelial tissue. In female patients, it seems to be an association between BMPM and endometriosis [3], pelvic inflammatory disease, and previous abdominal surgeries [4]. However, cases in men and children have been reported also. Due to the rarity of the disease, the pathogenesis and etiology of BMPM remains unclear [5]. We herein present a case of BMPM in a postmenopausal woman complicating with an ovarian cyst.

## Patient and observation

**Patient information**: a seventy-one-year-old woman presented to our outpatient department with a history of persistent low back pain. She had previously visited an orthopedic who suggested a lumbar spine computed tomography (CT). CT findings demonstrated abdominal cystic formations.

**Clinical findings**: on clinical examination, a palpable mass and mild tenderness in the lower abdomen were elicited; therefore, she underwent an abdominal Magnetic Resonance Imaging (MRI).

**Timeline**: after initial clinical assessment of the patient, the diagnosis was persumed to be a musculoskeletal condition. During evaluation, CT findings did not match with symptoms. Thus, an incidental finding of a multicystic intraperitoneal lesion was noticed and it was decided to perform an excision.

**Figure 1:**
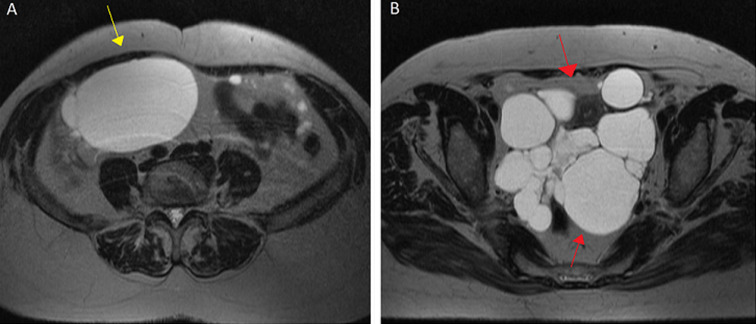
MRI axial scan shows: A) cystic lesion in the intraperitoneal subhepatic space (yellow arrow); B) multiple small cysts coexist in the perirectal fat (red arrows)

**Diagnostic assessment**: MRI revealed multiple cystic lesions (dimensions of 11 x 12 x 12, 5 cm) with thin septations but no papillary projections occupying the largest part of the pelvis, mainly behind the uterus. Multiple small cysts coexist also in the perirectal fat, pericolically and the greater omentum. Another lesion with similar characteristics (dimensions of 9,5 x 11, 5 x 15 cm) was observed in the intraperitoneal subhepatic space (Figure 1).

**Figure 2:**
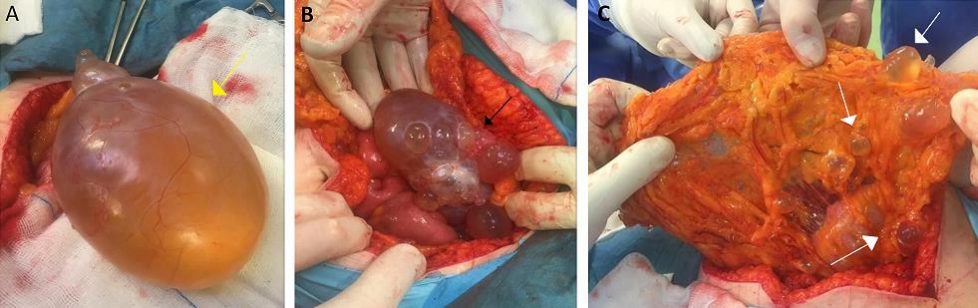
perioperative findings shows: A) large left ovarian cyst (yellow arrow) B. right ovarian cyst (black arrow); C) small cysts in the peritoneum (white arrows)

**Therapeutic intervention**: the patient was taken up for exploratory laparotomy after obtaining informed consent. Perioperative findings demonstrated free intraperitoneal clear fluid, a large left ovarian cyst as well as an accompanying ovarian cyst on the right side with smooth walls and multiple small cysts in the peritoneum, arranged in a grape-like form (Figure 2). Total hysterectomy with bilateral salpingo-oophorectomy, omentectomy and lymph node dissection were performed. Biopsies were taken from the peritoneum and both right and left paracolic gutters.

**Figure 3:**
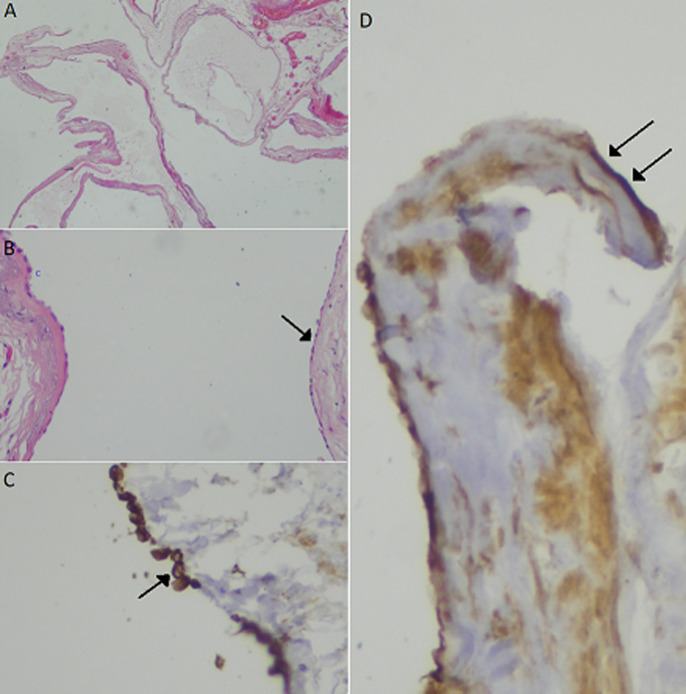
histopathologic findings; A): multiple cystic spaces without an obvious lining in this low power photo (20X); B) the lining is composed of flat cells, (arrow) and focally by cyboidal cells (small c) 100x; C): these lining cells show immunostaining for CKAE1/AE3 (single arrow); D): and for calretinin (double arrow)

**Diagnosis**: histopathology of the specimen revealed multiple cysts without malignant transformation suggesting a diagnosis of benign multicystic peritoneal mesothelioma (Figure 3).

**Follow-up and outcome of interventions**: the patient´s postoperative course was uneventful and she was discharged on postoperative day 5 with advice of regular follow-up.

**Patient perspective**: “I used to think that the low back pain was caused by a spinal problem. I felt really anxious when I heard about the CT findings. My doctor suggested to undergo a surgery. Since the histopathology revealed a benign neoplasm, I felt relief with the final diagnosis. My doctor reassured me and suggested annual follow-up. I hope everything will be fine”.

**Informed consent** was obtained.

## Discussion

Benign multicystic peritoneal mesothelioma was first described in 1928 by Plaut during an operation for uterine leiomyoma [2]. Due to the rarity of the tumor, the etiology and pathogenesis of BMPM remains unclear [6,7]. Given the higher incidence in women during their reproductive age, BMPM is tightly linked to sex hormones [8,9]. There seem to be an association between BMPM and pelvic inflammatory disease, endometriosis and previous abdominal surgeries; hence, some authors believe that BMPM derives from chronic inflammatory processes involving the peritoneum, which results in a reactive hyperplastic transformation of mesothelial cells [1,2]. Others suggest a more primitive neoplastic origin of the tumor with slow progressive growth but without strict association with previous inflammatory insult. Although the likelihood for transformation to malignancy is very small, there are some reported cases of malignant transformation, thus a long-term follow-up is considered essential [10-12].

The majority of patients with BMPM are asymptomatic and are diagnosed during a routine medical examination or in some cases as an incidental operative finding [6]. The most common symptoms occur mostly when the tumor is enlarged and include chronic abdominal and/or pelvic pain, abdominal distention and changes in bowel habits. A clinical examination may reveal abdominal or pelvic mass. Imaging methods such as ultrasound, computed tomography (CT) and magnetic resonance imaging (MRI) are needed to reveal the disease but a definitive diagnosis relies primarily on histopathological examination and immunohistochemistry [13].

The differential diagnosis mainly includes lymphangioma, pseudomyxoma peritonei and malignant peritoneal mesothelioma [14]. Lymphangioma consists of cysts with chylous fluid and lymphoid aggregates. On the other hand, malignant mesothelioma is associated with a history of exposure to asbestos, abdominal pain, ascites and weight loss. A plain chest radiograph may show signs of asbestos while an abdominal CT may reveal the presence of ascitic fluid and diffuse peritoneal thickening. It is well established that the optimal treatment strategy for BMPM is complete tumor resection in order to avoid recurrence. Because of its benign nature, chemotherapy and radiotherapy are not indicated for patients with BMPM. Other conservative treatments such as sclerotherapy, hormonal therapy (using anti-estrogens or gonadotropin-releasing analogues) and thermotherapy have provided uncertain therapeutic effects [14]. The correlation between rupture of a cystic lesion of BMPM and recurrence of disease is uncertain.

## Conclusion

Benign multicystic peritoneal mesothelioma is a rare cystic benign neoplasm and definitive diagnosis requires histopathological analysis. In postmenopausal women, BMPM should be included in the differential diagnosis when cystic tumors are identified in the anatomical area of the adnexa or pelvis. Complete resection of the tumor is the ideal treatment and malignant transformation is uncommon. Further studies are needed to better understand its pathogenesis and biological behavior.
